# Recurrent dysplasia epiphysealis hemimelica: a case report and review of the literature

**DOI:** 10.3389/fmed.2026.1750247

**Published:** 2026-05-25

**Authors:** Yakun Zhou, Dongfeng Chen, Ruotong Li, Yanchen Liang, Xiuming Miao

**Affiliations:** 1 The First School of Clinical Medicine, Shandong University of Traditional Chinese Medicine, Jinan, Shandong, China; 2 The Affiliated Taian City Centeral Hospital of Qingdao University, Tai’an, Shandong, China; 3 Orthopedic, Affiliated Hospital of Shandong University of Traditional Chinese Medicine, Jinan, Shandong, China; 4 Department of Pathology, Affiliated Hospital of Shandong University of Traditional Chinese Medicine, Jinan, Shandong, China

**Keywords:** case report, dysplasia epiphysealis hemimelica, pathology, surgical approach, Trevor’s disease

## Abstract

This paper presents a case of Dysplasia Epiphysealis Hemimelica that recurred 3 months following the initial surgical intervention. The recurrence was notably characterized by increased pain, limping, and significant ankle stiffness compared to the initial presentation. Histopathological analysis of the recurrence revealed immature fibrocartilaginous tissue, nodular proliferation of collagenous fibrous tissue, and cartilage fragments, accompanied by a foreign-body granulomatous reaction. A review of the literature suggests that recurrence of this condition is closely linked to incomplete surgical excision and the patient’s age. Based on these insights, we propose a postoperative follow-up protocol to mitigate the risk of recurrence.

## Introduction

Dysplasia Epiphysealis Hemimelica (DEH), also known as Trevor disease, is a rare, non-hereditary disorder of epiphyseal development characterized by focal osteochondral overgrowth arising from one or more epiphyses or ossification centers (1). Previous studies have estimated an incidence of approximately 1 per 1,000,000 individuals, with a higher prevalence in males. The condition typically presents during childhood and most commonly involves the lower extremities, particularly the knee, ankle, and foot (2, 3).

However, the clinical spectrum of DEH is not limited to lower-limb involvement in children. In recent years, several atypical presentations have been reported, including involvement of the upper extremities and rare cases with adult onset. Assan et al. described a case of DEH affecting the wrist and noted in their literature review that, although upper-limb involvement is uncommon, it has been documented (4). Similarly, Ocak and Çetin reported unusual manifestations of DEH and emphasized that the condition should be considered in the differential diagnosis even when lesions occur at atypical anatomical sites or in atypical age groups (5).

Because DEH is extremely rare and its clinical and radiological features may overlap with those of osteochondroma and other osteochondral lesions, misdiagnosis or delayed diagnosis may occur in clinical practice. Improving recognition of its clinical manifestations, imaging characteristics, and key points in differential diagnosis is essential for achieving early and accurate diagnosis and appropriate management. Although surgical excision generally yields favorable outcomes, postoperative regrowth or recurrence may occur, and reports describing early recurrence remain limited.

In this context, we report a rare case of ankle DEH in which the lesion recurred within 3 months after surgical excision. In conjunction with a focused review of the literature, we discuss the diagnostic reconsideration process, the differential diagnosis with osteochondroma, treatment strategies, and implications for postoperative follow-up. This report aims to enhance clinical awareness of atypical presentations of DEH and highlight considerations regarding early recurrence. The case report was prepared in accordance with the CARE guidelines (Supplementary Material 1).

## Case report

We present the case of a 9-year-old female patient whose mother delivered her naturally at the age of 41. The pregnancy was routinely monitored through prenatal checkups, which revealed no significant abnormalities, complications, or comorbid conditions. Labor and delivery were uneventful, with no instances of prolonged labor, postpartum hemorrhage, or other adverse events. Postnatal physical examination showed no congenital malformations, and the patient’s growth and developmental indicators were within normal ranges. Her medical history was unremarkable, with no notable illnesses or accidental injuries during childhood.

**FIGURE 1:**
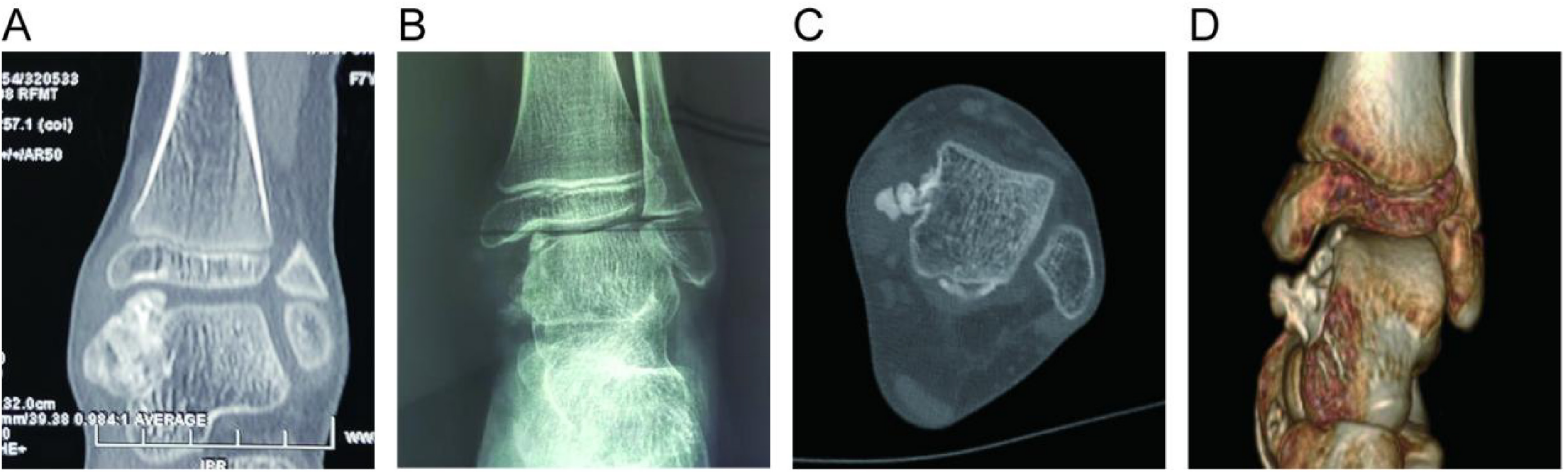
**(A)** Initially, a CT plain scan reveals an irregular mass of diffusely proliferating tissue in the left talus. The texture is uneven, and the posteromedial bony cortex of the talus appears blurred, exhibiting signs of destruction. **(B)** Following the first operation, a digital radiograph (DR) shows a cloud-like shadow at the medial margin of the talus. **(C,D)** On the recurrent CT plain scan and three-dimensional reconstruction, an irregularly shaped elevation with inconsistent bone density is observed at the medial margin of the talus, presenting a lobulated appearance. Its volume is smaller compared to the initial onset.

**FIGURE 2:**
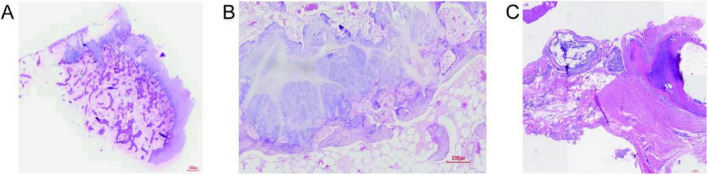
**(A)** Shows the pathological histological morphology of the lesion in the first surgery. The surface of the lesion was rough and uneven, without a fibrous membrane covering. The cells in the cartilage cap were irregularly arranged, mainly consisting of chondrocytes in the mature and hyperplastic zones, and cartilage islands could be seen. Endochondral ossification was observable. **(B)** Depicts the chondrocytes in the hyperplastic zone of the lesion, which were distributed in columns and clusters. Some cells showed mild atypia, and no mitotic figures were present. **(C)** Represents the scraping specimen from the second surgery: It consisted of immature fibrocartilaginous tissue, nodularhyperplastic collagen fiber tissue, and cartilage fragments, accompanied by a foreign body granulomatous reaction in the surrounding area.

At the age of 8, a painless swelling over the ankle was incidentally discovered during bathing, accompanied by noticeable limping while running. The patient was evaluated at a local hospital, where X-ray and CT imaging identified a tumor in the left talus (Figure 1A). Surgical resection was performed (Figure 1B), and postoperative histopathology confirmed the presence of osteochondroma. Microscopic examination revealed a rough and irregular lesion surface, lacking a fibrous membrane covering. The cartilage cap cells were irregularly arranged, predominantly consisting of chondrocytes resembling those of mature and proliferative zones, distributed in columnar and clustered patterns. Mild cellular atypia was noted, with no evidence of mitotic figures. The calcified zone was thin and irregular, containing scattered cartilage islands and areas of endochondral ossification (Figures 2A,B). The final clinical diagnosis was osteochondroma, and the patient was discharged post-surgery.

Three months postoperatively, follow-up X-ray imaging demonstrated a smaller bony protrusion at the medial border of the talus, suggesting recurrence. The patient reported pain and limping during ambulation, with increased ankle stiffness compared to the initial onset. She was referred to our hospital, where further CT and 3D reconstruction revealed an irregularly contoured osseous mass with a lobulated appearance at the medial-superior region of the left talus, accompanied by swelling of surrounding soft tissue. Based on the imaging findings and the short-term recurrence, hemimelic epiphyseal dysplasia was suspected. According to the clinical classification proposed by Azouz, the lesion was categorized as type I (6). A medial malleolar turnover osteotomy with curettage of the lesion was planned (Figures 1C,D).

During surgery, an anteromedial approach to the ankle was utilized. The skin, subcutaneous tissue, and deep fascia were incised sequentially to expose the tibio-talar joint cavity. Exploration revealed abundant gelatinous, mucinous degenerative tissue in the joint cavity and evidence of articular cartilage destruction on the talus, without fracture or bone bridge formation but with mild synovial exudation. In accordance with the family’s preference, medial malleolar osteotomy was not performed, limiting visualization of the posteromedial region and preventing en bloc tumor resection. Consequently, only limited intralesional curettage of the anterior portion of the lesion and removal of gelatinous tissue were performed, which were submitted for paraffin-embedded pathological evaluation to guide potential reoperation or adjunctive therapy.

Postoperative pathological examination of the submitted specimen (talus bone tumor, left side) revealed a small amount of osseous tissue, immature fibrocartilage, and nodularly proliferating collagenous tissue. Multiple foci of cartilage fragments were observed within the bone marrow cavity and collagen fibers, accompanied by surrounding foreign-body granulomatous reactions (Figure 2C).

At the patient’s initial presentation, the condition was misdiagnosed. The patient subsequently underwent lesion excision; however, recurrence was detected three months postoperatively during follow-up. Three-dimensional reconstruction at the current evaluation revealed that the majority of the tumor was located in the posteromedial aspect of the talus, with its anterior margin obscured by the deep fibers of the deltoid ligament. Accordingly, a medial malleolar osteotomy was recommended to provide adequate exposure and facilitate as complete a resection as possible. However, the patient’s family expressed substantial concern regarding potential complications associated with osteotomy and its possible impact on ankle joint function, and therefore declined the extended resection strategy. After thorough discussion, only partial tumor resection was ultimately performed. Telephone follow-up after surgery indicated that the patient currently experiences no significant pain and reports no obvious limitations in daily activities. Although the family is generally satisfied with the current symptomatic relief, they declined further outpatient visits and imaging follow-up, precluding the acquisition of more comprehensive objective functional assessments and imaging data.

## Literature review

### Materials and methods

This study systematically searched the PubMed, Cochrane Library, and Web of Science databases. The search terms included “Dysplasia Epiphysealis Hemimelica,” “Trevor disease,” and “case report” (Supplementary Material 2). The search covered the period from January 1, 2016, to April 6, 2026. Both free-text terms and controlled vocabulary were used, and no language restrictions were applied. In total, 39 articles involving 45 patients were included. The following data were extracted from each study: first author and year, sex, age of onset (years), lesion site, clinical manifestations, imaging findings, pathohistological features, treatment, follow-up duration (months), and prognosis (Table 1).

**TABLE 1:** Information of included literature.

First author and year	Gender	Age at onset (years)	Lesion site	Clinical manifesta-tions	Imaging findings			Pathohisto-logical features	Treat-ment	Follow-up duration (months)	Prognosis	Reference
					X-ray	CT	MRI					
Gregory Benes 2023	M	9	Hip	Limp, restricted ROM	Mass lesion in the right acetabulum causing right hip subluxation, right hip dysplasia, increased acetabular index, and blunting of the lateral sourcil.	Intra-acetabular lesion of the right hip.	Osseous abnormality at the base of the right acetabulum extending to the triradiate cartilage, approximately 5.5 × 2.5 cm, confluent with the acetabular base.	Osteochondroma with a variably thick cartilage cap.	Open excision	4	Good	(7)
Bo Li 2019	F	9	Talus	Pain, mass	Smooth-margined osseous mass at the right medial talus.	Osseous masses in the right talus and medial talar region with heterogeneous bone density and marked sclerosis; pseudoarthrosis was visible between the lesion and the talus.	Detached mixed-signal lesion in the right talus and medial talar region with marked sclerosis.	Abundant fibroblasts and cartilaginous tissue, without atypia.	Open excision	6	Good	(8)
Anil KC 2022	M	11	Hip, femur	Restricted ROM	Huge irregular epiphyseal mass in the right hip with extensive ossification.	Marked enlargement and deformity of the right femoral head measuring 14 × 12 × 10 cm, extending into the acetabulum and joint cavity.	NA	Chondroma-like features.	Open excision	12	Good	(9)
Saurabh Vashisht 2020	M	11	Patella	Pain, mass, restricted ROM	Osseous prominence at the superior pole of the right patella with associated mass formation and focal calcification.	Bony prominence at the superior pole of the patella with a large mass containing focal calcified areas.	The mass showed low-to-intermediate signal on T1-weighted images and iso- to high signal on T2-weighted images, suggesting cartilaginous content.	The cartilage cap was composed of hyaline cartilage; endochondral ossification with trabecular bone formation was seen at the base of the cap.	Open excision	1	Good	(10)
Kemal Gökkuş 2017	M	23	Tibia	Pain, restricted ROM	Spur-like protrusion at the anterior aspect of the distal left tibia.	Anterior bony spur or residual small osseous protrusion at the distal tibia.	Spur-like lesion with bone marrow edema and perilesional synovial effusion.	Osteochondroma-like appearance.	Arthroscopic excision	2	Good	(3)
	M	13	Tibia, fibula	Pain, mass, restricted ROM	Irregular small chondroma-like mass anterior to the right ankle.	Irregular osseous granules (coalescent ossified fragments) at the lateral distal tibial metaphysis; a cartilage cap was visible over the bone.	NA	NA	Open excision	9	Good	
	M	12	Calcaneus	Pain, mass, restricted ROM	Lobulated ossified mass in the posterior calcaneal region.	NA	NA	NA	Conservative management	NA	No obvious deformity or gait disturbance	
	M	4	Femur, tibia	Mass, limp, deformity	Osteochondroma-like osseous mass at the posteromedial ankle; epiphyseal overgrowth of the lateral distal femur. Involvement of the lateral half of the femoral head epiphysis was also observed.	NA	NA	NA	Open excision + epiphysiodesis	6	Good	
Suhaib Bani Essa 2025	F	8	Carpal bones	Mass, restricted ROM	Irregular ossified lesion on the dorsal aspects of the right scaphoid, capitate, and trapezoid.	Ossification and cartilaginous overgrowth over the dorsal scaphoid, capitate, and trapezoid.	Osteochondral overgrowth of the scaphoid and trapezoid.	Cap of mature hyaline cartilage over mature bone with overlying fibrocartilaginous perichondrium.	Open excision	36	Good	(11)
Maurizio De Pellegrin 2024	M	2	Talus	Pain	Posterior ossification center of the left talus.	NA	Newly formed body with an osteochondral architecture and a prominent cartilaginous component.	Surface cartilaginous proliferation with trabecular bone and marrow spaces internally.	Open excision	12	Good	(12)
Fırat Ozan 2016	F	9	Talus	Pain	Localized irregular osseous mass at the anteromedial talus and medial tibial side.	Lobulated osteochondral mass at the anteromedial talus and medial distal tibia.	NA	Surface layer of hyaline cartilage, columnar chondrocytes, and mature trabeculae containing bony spicules and marrow.	Open excision	14	Good	(13)
Adelina Ionescu 2021	M	8	Talus	Pain, mass, restricted ROM, deformity	Multiple heterogeneous ossification centers around the right talus anteriorly, posteriorly, and medially, resembling osteochondromas and separated from the talus and distal tibial epiphysis; deformity of the medial tibial articular surface.	Multiple centers of endochondral ossification adjacent to the right talus.	Periosteal edema and delineation of the relationship between the tumor mass and adjacent soft tissues.	Tissue fragments composed of bone with surface cartilaginous proliferation; minimal atypia, irregular distribution, limited basal columnar arrangement, mildly increased cellularity, and mineralized areas within zones of endochondral ossification. Fibrous connective tissue was observed on the surface of the cartilaginous proliferation (perichondrium).	Open excision	1	Good	(2)
	M	8	Talus	Deformity	Bony mass composed of multiple ossification centers adjacent to the left talus and lateral malleolus, extending posteriorly and arising from the talus.	NA	NA	NA	Conservative management	NA	No progression	
Hiva Mohamadian 2021	M	10	Radius	Restricted ROM	Irregular mass with focal ossification in the proximal radial epiphysis.	Irregular mass with focal ossification in the proximal radial epiphysis.	Epiphyseal cartilaginous lesion at the radial head.	Clusters of chondrocytes in a fibrous stroma, with a thick cartilage cap and ossification centers.	Open excision	6	Good	(14)
Clément Jeandel 2021	M	11	Femur, patella	Pain, limp	Multiple free calcified loose bodies within the left knee.	Superficial osteochondromas involving the medial femoral condyle and patella.	Multiple large intra-articular loose bodies and two osteochondral-signal lesions, one arising from the medial femoral condyle and one from the patella.	Osteochondroma-like appearance.	Open excision	60	Good	(15)
Malik I. Ali 2019	M	16	Carpal bones	Pain, restricted ROM	Osseous abnormality on the volar surface of the lunate.	The lesion abutted the adjacent scaphoid and triquetrum.	A 1 × 2 × 1.1 cm lesion in the volar carpal region with peripheral calcification.	Mature cartilage and bone with mildly disorganized endochondral ossification at the interface.	Open excision	12	Good	(16)
Adam Bacon 2022	F	4	Femur	Pain, mass, deformity	Calcified mass at the posteromedial distal right femur.	NA	Osteochondral mass at the medial posterior femoral condyle; the cartilaginous component contained mature osseous foci discontinuous from the adjacent normal epiphysis.	NA	Arthroscopic exploration + open excision	84	Good	(17)
Gholam Hossain Shahcheraghi 2020	M	1	Femur, tibia, talus	Mass, restricted ROM, deformity	Genu valgum with large lesions visible around the knee and ankle.	NA	NA	NA	Open excision + tibiofibular osteotomy; repeat open excision + proximal tibial varus/internal-rotation osteotomy; physeal arrest at age 8 years.	36	Recurrence several months after the first operation, recurrence 2 years after the second operation, and no obvious regrowth after the third operation.	(18)
Gholam Hossain Shahcheraghi 2017	M	6	Femur, tibia, talus	Limp, restricted ROM, deformity	Hip radiograph showed an almost “inverse ball-and-socket” or “flat-on-flat” joint, with a prominent acetabulum, huge deformed femoral head, and narrowed joint space; ankle radiograph showed talar and tibial deformity with multiple massive osteophytes and subtalar fusion.	NA	NA	NA	Arthroplasty	30	Good	(19)
Spandan Koshire 2022	M	8	Tibia, talus	Pain	A 1.5 × 1 cm osseous fragment at the ankle with irregular cortical thickening.	Symmetric bony overgrowth of the medial distal tibial epiphysis with talar protrusion.	Osseous fragments arising from the tibial plafond and anteromedial talus, without associated periosteal soft-tissue abnormality or periosteal reaction.	NA	Open excision	3	Good	(20)
Amrit S Khalsa 2017	F	39	Tibia	Pain	Abnormal calcific density above the anterolateral tibial plateau.	Exophytic lobulated sclerotic mass measuring 2.4 × 0.9 × 0.8 cm involving the anterior margin of the lateral tibial plateau.	A 1.2-cm well-circumscribed lesion along the anterolateral cortical surface of the tibial plateau.	Sclerotic bone with overlying cartilage.	Open excision	30	Recurrence 2.5 years after the first operation	(21)
Fei Gao 2020	M	50	Femur	Pain, mass, deformity	Asymmetric enlargement of the medial epiphyseal cartilage of the distal femur.	NA	NA	NA	Arthroplasty	24	Good	(22)
Ruken Yuksekkaya Celikyay 2016	M	3.5	Femur, tibia	Mass, deformity	Marked asymmetric cartilaginous/ osseous overgrowth of the left hip, knee, and ankle epiphyses, particularly irregular exophytic ossification at the lateral femoral epiphysis.	Osseous overgrowth along the lateral femoral and tibial epiphyses.	Asymmetric osteochondral mass continuous with the distal femoral and proximal tibial epiphyses. The cartilaginous component showed intermediate signal intensity on fat-suppressed proton-density images and low signal on T1-weighted images.	Chondrocytes clustered in the deep layer of hyaline cartilage with clefts extending into the articular cartilage.	Open excision	6	Recurrence 6 months postoperatively	(6)
Timothy W. Torrez 2021	F	7	Humerus	Restricted ROM	Large (3.2 × 1.7 cm) exophytic mass posterior to the humeral epicondyle, extending distally to the capitellum.	Mass connected to the posterolateral humeral cortex, with punctate calcifications in the ulnohumeral joint.	NA	NA	Open excision	4	Good	(23)
Takeo Mammoto 2018	M	9	Femur	Pain	Irregular exophytic ossification at the posteromedial femur.	Lobulated irregular osseous hypertrophy at the posteromedial femur.	Asymmetric osteochondral lesion continuous with the distal femoral epiphysis, with swelling of the articular surface and cartilage bulging.	NA	Arthroscopic excision	3	Good	(24)
Tarek N. Fetih 2019	M	6	Talus	Pain, mass	Irregular talar articular surface with extensive calcification in the anterior joint compartment and posterior talus.	Sheet-like irregular osseous protrusions above the talus with narrowing of the tibiotalar joint space.	Bone marrow edema adjacent to the osseous protuberance, with synovitis and a small amount of joint effusion around the lesion.	NA	Open excision	6	Good	(25)
Mandeep Singh Dhillon 2022	M	9	Tibia, talus	Pain, mass	Two exostotic lesions along the distal tibia and superior aspect of the talus, predominantly cartilaginous.	Exophytic osseous lesions arising from the tibia and talus.	NA	NA	Open excision	8	Good	(26)
Bo Zhang 2021	M	6	Tibia, talus	Mass, limp, restricted ROM	A 2.2-cm exophytic lesion with a distinct cortex located at the anteromedial aspect of the right ankle.	NA	Anteromedial ankle exostosis without articular damage, with bony continuity to the tibia or talus.	Osteocartilaginous proliferation.	Open excision	12	Good	(27)
Esat Uygur 2018	M	6	Tibia, talus	Pain, mass	Multiple atypical osteochondromas involving both the talus and distal tibial metaphysis.	The medial and anterolateral talus and the tibiotalar joint were involved.	No other musculoskeletal involvement was detected.	NA	Open excision	12	Good	(28)
Nikolaos A. Stavropoulos 2026	M	17	Humerus	Pain, mass	Mass-like, bilobed, symmetric osteochondral overgrowth of the proximal humeral epiphysis with stippled calcifications and cortical thinning.	Extensive cartilage calcification, cortical remodeling, and an osteolytic area with cortical destruction posterior to the epiphyseal overgrowth.	A large lobulated medial compartment lesion extending to the cortex, showing low signal intensity on T1-weighted images and high signal on fat-suppressed T2-weighted images, consistent with hyaline cartilage.	Low-grade cartilaginous tumor with close osseous infiltration; lobules of hyaline cartilage extended between cancellous trabeculae into the periosteal soft tissue.	Arthroplasty	42	Good	(29)
Daine O. Clarke 2016	F	7	Femur	Mass, deformity	Calcified mass posterior to the femur.	NA	A 3.2 × 2.9 × 3.5 cm epiphyseal mass protruding toward the popliteal fossa.	NA	Open excision	48	90° fixed flexion deformity of the left knee and rigid equinovarus deformity of the left ankle	(30)
Shinsuke Sato 2021	M	7	Talus	Pain, mass, restricted ROM	Multiple oval loose bodies, 10–15 mm in diameter, in the anterior and posterior ankle recesses.	Multiple intra-articular ossified oval masses and several protrusions arising from the talus.	Intra-articular oval masses showed low signal on T1-weighted images and partially high signal on T2-weighted and STIR images. Diffuse STIR high-signal changes were also present in the talar protrusion and adjacent talar body, suggesting marrow involvement.	No hyaline cartilaginous component was identified in the synovium. The brown mass consisted of trabecular bone almost entirely covered by fibrous tissue, with only scant cartilage. The white masses were loose bodies composed of thick hyaline cartilage (cartilage cap) with central ossification, resembling epiphyses; chondrocyte clusters were present, and the central osseous tissue was largely necrotic. No sarcomatous component was found.	Open excision + arthroscopic excision	10	Recurrence 10 months after the first operation, with ankle locking and new oval ossified loose bodies in the posterior recess of the left ankle on radiographs	(31)
John M. Kopriva 2019	F	6	Radius	Pain, restricted ROM	Malalignment of the distal right radius with loss of radial inclination; signs of bony bridging.	Osseous proliferation from the distal radial epiphysis toward the distal radioulnar joint.	Osseous proliferation from the distal radial epiphysis toward the distal radioulnar joint.	Endochondral bone formation with mature bone in the deeper portion.	Open excision	30	Good	(32)
	F	8	Radius, ulna	Restricted ROM	Spherical enlargement and mild shortening of the distal ulna, bridging the distal radioulnar joint.	NA	Dysplastic osteochondral growth at the distal radius extending toward and partially overlapping the ulna.	NA	Conservative management	8	No pain or limitation of motion	
Hasan Ocak 2022	M	54	Humerus	Pain, restricted ROM	Marked degenerative changes in both shoulder joints with substantial destruction of the oval structures of both humeral heads.	NA	Flattened humeral heads with marked bilateral osteoarthritis, synovitis, and loose bodies.	NA	NA	NA	NA	(5)
Vincenzo Giordano 2019	M	58	Humerus	Pain	Abnormal morphology of the left humeral head, with an anteromedial exostotic recess arising from the epiphysis.	Ossified mass at the inferomedial aspect of the proximal humeral epiphysis, without joint or soft-tissue involvement.	Left shoulder T1-weighted, T2-weighted, and STIR images showed an inferomedial mass protruding into the dependent axillary recess, with sharpening of the humeral head articular cartilage but no evidence of osteoarthritis.	NA	Conservative management	NA	NA	(33)
Prabodh Kantiwal 2026	F	4	Carpal bones	Pain, mass, deformity, restricted ROM	Multiple expansile, densely sclerotic, multilobulated lesions with irregular margins involving the scaphoid, lunate, and trapezium.	Expansile, sclerotic, “popcorn-like” bony architecture within the scaphoid, lunate, and trapezium.	A heterogeneous adherent lesion arising from the carpal bones, with a smaller lesion near the scaphoid extending to the abductor pollicis longus tendon sheath and characterized by a low-signal cartilaginous rim.	Cartilage cap covering mature trabecular bone.	Open excision	NA	NA	(34)
Juan J. Dominguez-Amador 2018	M	12	Radius	Pain, mass, deformity, restricted ROM	Alteration of the distal radial epiphysis with dorsal bony overgrowth; the lesion was irregular and contained internal calcifications.	Overgrowth of the distal radial epiphyseal ossification center measuring approximately 18 × 18 × 23 mm.	NA	NA	Open excision	9	Good	(35)
Cosma Calderaro 2017	M	9	Talus	Pain, restricted ROM	Irregular bony prominences localized to the anteromedial and posteromedial talus.	Bony protrusions at the anteromedial and posteromedial talus; ankle joint space preserved.	Superior-medial talar osseous prominence with ankle edema.	NA	Open excision	60	Good	(36)
	M	10	Talus	Pain	Leaf-like osseous prominence arising from the medial articular portion of the talus, with a large cauliflower-like loose body within the joint.	Lesion arising from the anteromedial talus with irregularity of the distal one-third of the tibia.	Pedunculated oval osseous tumor arising from the talus.	Cartilaginous mass without evidence of tumor cells.	Arthroscopy + open excision	6	Good	
Nickolaos Laliotis 2023	M	4	Talus	Mass	Calcified mass at the posterior and medial ankle. The distal tibial epiphysis and the contours of the talus and calcaneus were normal. Calcifications were uniformly distributed within the mass.	The lesion was largely calcified; a small calcified stalk was seen between the lesion and the talus, but no clear continuity was identified.	Heterogeneous mass adjacent to the posterior talar margin with ankle edema.	Mature cartilage at the periphery with central cancellous bone.	Open excision	3	Good	(37)
Samuel S. Y. Wang 2019	M	18	Femur	Pain, restricted ROM	Joint displacement with an irregular epiphyseal mass showing extensive intracapsular ossification.	Delineated the extent of femoral head involvement.	NA	NA	Arthroplasty	NA	Good	(38)
Kintzelé L 2016	M	8	Tibia, talus, navicular, medial cuneiform	Pain, deformity	The distal tibial epiphysis narrowed progressively from medial to lateral; multiple small calcified structures were visible in the projection of the distal tibial epiphyseal plate and superior ankle joint, with additional ossified structures posterior to the ankle.	Multiple cartilage-derived ossified structures, some extending into the joint, located in the distal tibial epiphysis, talus, navicular, and medial cuneiform.	NA	NA	Conservative management	12	Marked enlargement of the ossified structures	(39)
Chia-Hui Chen 2025	M	9	Humerus	Restricted ROM, upper-limb length discrepancy	Multiple ossified masses inferior to the humeral head and related to the glenoid.	NA	Intra-articular masses with multiple focal ossification centers involving the right humeral head and glenoid, accompanied by epiphyseal cartilaginous overgrowth.	NA	NA	NA	NA	(40)
Kosuke Sumi 2025	M	9	Hip	Pain, restricted ROM, limp	Right hip subluxation.	NA	Continuous protruding lesion from the acetabular floor, showing high signal on T2-weighted imaging.	Osteochondral tissue with a thick, irregular cartilage cap and discontinuous ossification centers.	Arthroscopic excision	24	Good	(41)

Inclusion criteria:

(1) Patients with a confirmed diagnosis of DEH/Trevor disease;
(2) Article types limited to case reports or case series;
(3) Availability of full-text articles with extractable core data;
(4) In cases of duplicate reports, only the most comprehensive or most recently published article was included.

Exclusion criteria:

(1) Studies unrelated to DEH/Trevor disease or with unclear diagnoses;
(2) Review articles, systematic reviews, meta-analyses, expert commentaries, editorials, conference abstracts, or articles with abstracts only and no full text;
(3) Basic research or technical reports lacking extractable individual patient data;
(4) Studies with severely incomplete clinical data;
(5) Duplicate publications or repeated case reports.

### Results

Among the patients, 35 were male (77.78%) and 10 were female (22.22%). The mean age was 12.41 years [standard deviation (SD) 12.81; range, 1–58 years]. Multiple lesions were observed in 28.89% of cases. Considering all reported lesion sites, the talus was the most commonly affected bone (17 cases), followed by the tibia (12 cases) and femur (11 cases). Less frequently involved sites included the humerus (5 cases), radius (4 cases), hip (3 cases), carpal bones (3 cases), and patella (2 cases). Single cases involving the fibula, ulna, and calcaneus were also reported.

**FIGURE 3:**
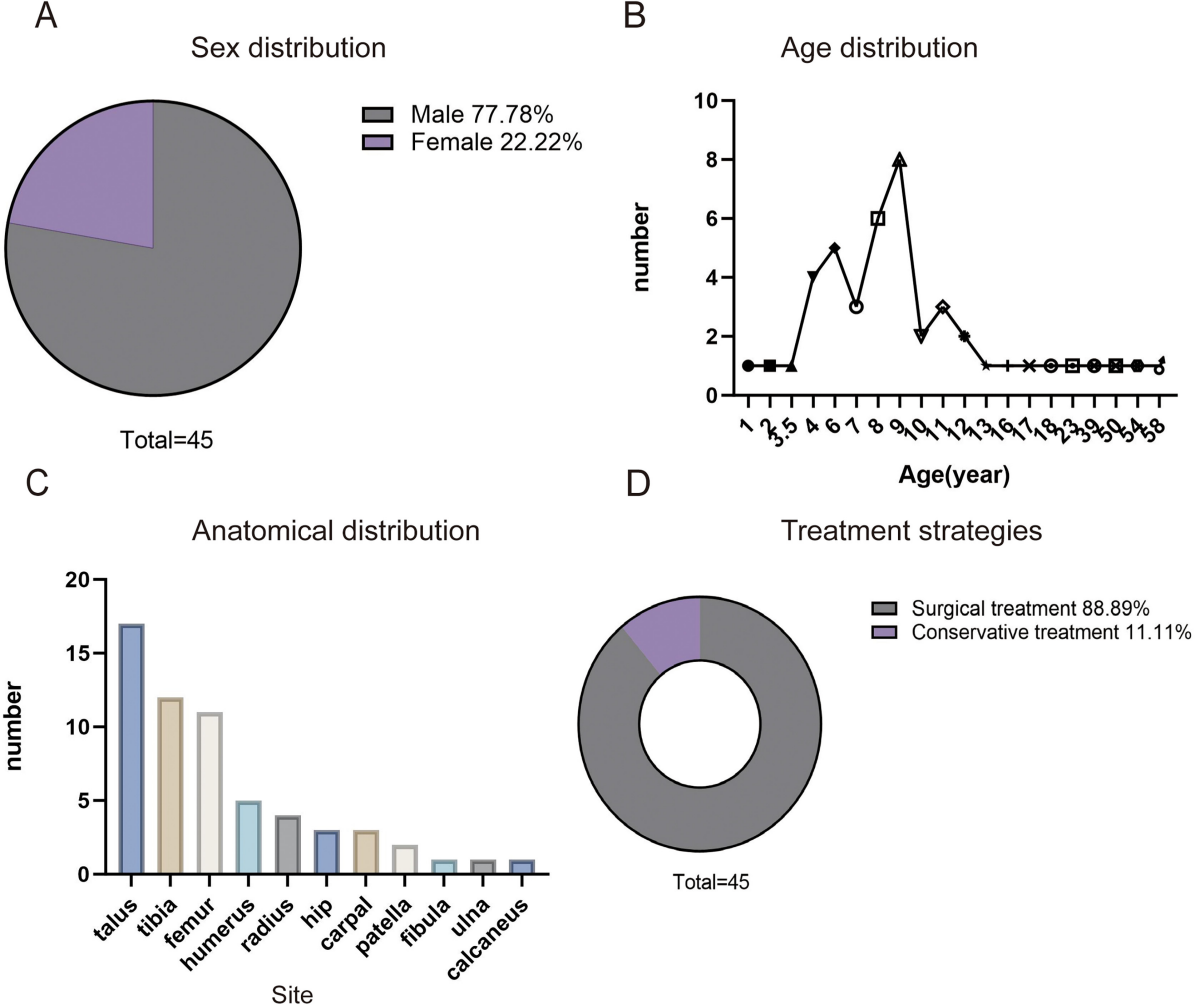
**(A)** Sex distribution—35 males and 10 females. **(B)** Age distribution—age at onset was primarily between 4 and 9 years. The youngest patient was 1 year old and the oldest was 58 years. **(C)** Anatomical distribution—lesions most commonly occurred in the lower extremities, particularly in the talus (17 cases). In contrast, involvement of the upper extremities was less frequent, with the humerus being the most commonly affected site (5 cases). **(D)** Treatment strategies—management was predominantly surgical, with 40 cases undergoing surgical treatment.

According to the literature, pain was the most common clinical manifestation, occurring in approximately 66.67% of cases, while 46.67% of patients presented with a palpable mass. DEH caused limitation of motion in 53.33% of cases, deformity in 26.67%, and limping in 15.56%; two patients also exhibited limb-length discrepancy. Surgical treatment was performed in 88.89% of patients, including open excision, arthroscopic excision, and joint replacement, whereas 11.11% were managed conservatively. Postoperative recurrence was reported in four cases, and one patient developed postoperative joint stiffness and deformity (Figure 3).

## Discussion

The diagnostic process in this case evolved from an initial diagnosis of osteochondroma to a revised diagnosis of DEH. We consider this change to reflect an initial misdiagnosis resulting from limited recognition of this rare condition and difficulty in determining the precise origin of the lesion at the first visit, rather than a biological transformation of an osteochondroma into DEH during follow-up. Although DEH and osteochondroma can both present as osteochondral proliferative lesions and share certain histological features, they represent distinct disease entities (42). Osteochondromas typically arise from the metaphysis, whereas DEH primarily originates from the epiphysis or secondary ossification centers. The former generally appears as a common benign exophytic lesion on the bone surface, whereas the latter more often manifests as asymmetric epiphyseal overgrowth around joints in children (2). In the present case, reassessment of the two sets of imaging studies clarified the relationship between the lesion and the epiphysis. Moreover, the current pathological findings, considered together with the clinical and radiological features, further support the diagnosis of DEH. Therefore, this case is best interpreted as a process of diagnostic clarification through serial evaluation rather than a transformation in the biological nature of the lesion.

On the other hand, because the boundary between DEH lesions and normal epiphyseal cartilage is often indistinct, and because the initial surgery may have been limited in lesion exposure and completeness of excision, it is difficult to definitively distinguish between true recurrence and continued growth of residual disease when the lesion was detected again 3 months postoperatively. Based on the imaging findings and the limitations of the initial surgical approach, the posteromedial lesion is more likely to represent residual tumor from the first operation. In contrast, the anterior lesion was more closely associated with the patient’s pain symptoms, and its histological features demonstrated a degree of structural heterogeneity and disorganization, suggesting the possibility of early recurrence. Nevertheless, this interpretation remains speculative and should be approached with caution.

### Diagnosis and differential diagnosis

#### Symptoms and signs

DEH is typically diagnosed in early childhood. Initially, it often presents with pain, a palpable mass, and a limping gait. As the disease progresses, deformities such as varus or valgus angulation and limb length discrepancies may develop. A review of the literature indicates that pain is the most common clinical manifestation of DEH. However, in the present case, the patient did not experience significant pain at the onset; instead, the primary symptoms were a limp during running and a mass around the ankle joint. Noticeable pain emerged 3 months following the initial surgical intervention.

#### Imaging findings

Imaging examinations play a crucial role in both the diagnosis of DEH and therapeutic decision-making (43). Radiography typically demonstrates lesions arising from one side of the epiphysis, characterized by asymmetric enlargement of the epiphysis, multiple irregular ossification centers, and eccentric osteochondral-like proliferations around the joint. CT is useful for delineating the extent of the lesion and its osseous anatomical relationships, whereas MRI is better suited for evaluating unossified cartilage components, joint involvement, the condition of the growth plate, and the surrounding soft tissues. Previous studies have suggested that radiography serves as the primary modality for imaging evaluation of DEH, while CT mainly provides supplementary anatomical information for preoperative planning (8).

Osteochondroma represents the most important differential diagnosis. Unlike DEH, which originates from the epiphysis, osteochondroma typically arises from the metaphysis. Its key imaging feature is continuity of the lesion with the cortex and medullary cavity of the parent bone (44). CT can clearly demonstrate this continuity, whereas MRI is more suitable for evaluating the cartilage cap and adjacent soft tissues (45). Therefore, the principal distinction between these two entities lies in the site of lesion origin and the pattern of structural continuity.

#### Histopathological analysis

From a histopathological perspective, dysplasia epiphysealis hemimelica (DEH) is difficult to distinguish from osteochondroma. DEH typically manifests as osteocartilaginous proliferation with varying degrees of endochondral ossification, and its histological appearance is often described as an “osteochondroma-like lesion.” Consequently, pathological morphology alone is usually insufficient for reliable differentiation between the two entities; accurate diagnosis requires comprehensive evaluation incorporating clinical presentation and imaging findings, particularly the epiphyseal origin of the lesion (42). Previous studies have suggested that DEH and osteochondroma are not entirely identical in their morphological and immunohistochemical features; however, no widely accepted histological criteria currently exist that can independently establish the diagnosis (46, 47).

In the present case, histological findings differed between the two surgical specimens. The first specimen predominantly exhibited osteochondroma-like features, whereas the second was characterized mainly by scant osteogenic tissue, immature fibrocartilaginous tissue, and nodular proliferative collagenous fibrous tissue, accompanied by multifocal cartilage fragments and surrounding foreign-body granulomatous reactions. We speculate that, in addition to postoperative reactive fibrocartilaginous changes, the second recurrent specimen may represent a relatively early stage of recurrence, with histopathological features demonstrating structural diversity and disorganization. As the lesion progresses, cartilage islands may undergo maturation and ossification, eventually developing histological characteristics resembling osteochondroma. However, this interpretation remains a hypothesis based on the clinical course, serial imaging findings, and the pathological differences observed between the two specimens, rather than definitive evidence. Given the limited number of reported cases of recurrent DEH, the early pathological characteristics of recurrent lesions and their potential histological evolution require further clarification through the accumulation of additional cases.

### Treatment strategies

The management of DEH is generally divided into conservative and surgical approaches. Due to the condition’s rarity, there are currently no definitive surgical guidelines or standardized treatment algorithms. Elena Artioli and colleagues conducted a review of 70 cases, finding that 92% were treated surgically while 8% received conservative treatment. Mass excision was performed in 95% of the surgical cases, including seven that were done arthroscopically. The surgical techniques reported included osteotomy, microfracture, talonavicular arthrodesis, Achilles tendon lengthening, application of a Taylor Spatial Frame, medial distal tibial hemiepiphysiodesis, and phenol chemical curettage, among others (1). A review of the literature indicates that the condition does not carry malignant potential (48).

Asymptomatic patients may be managed conservatively with regular follow-up. In contrast, those presenting with symptoms such as pain, limping, restricted range of motion, or limb-length discrepancy may be candidates for surgical excision. Regarding surgical options, extra-articular lesions may be directly excised. For intra-articular lesions, arthroscopic evaluation is recommended. If the lesion maintains joint congruity, it may be retained and the deformity addressed with epiphysiodesis or osteotomy. However, incongruent lesions are best excised. In adult patients, joint arthroplasty may be considered.

### Prognosis

In terms of prognosis, DEH is generally considered a benign lesion; however, postoperative recurrence may occur, necessitating long-term follow-up. A retrospective study covering the period from 1970 to 2017 reported 28 cases of DEH from two hospitals, among which three experienced recurrence (49). Because of the rarity of this condition, reports of recurrence remain limited, and the underlying causes have not been thoroughly investigated. The authors suggested that recurrence may be associated with incomplete surgical excision and the growth potential of pediatric patients.

Regarding the mechanism of recurrence, incomplete resection is the most plausible explanation in this case. DEH is characterized by abnormal proliferation of epiphyseal cartilage, forming multicentric calcified or ossified masses that have indistinct boundaries with normal epiphyseal tissue. If pathological cartilage is not completely removed during surgery, residual active chondrocytes may continue to proliferate, leading to recurrence. In addition, the substantial growth potential in children may also contribute to regrowth. Shahcheraghi et al. reported a case involving a 4.5-year-old child in whom lesions in the knee and ankle could not be controlled despite repeated resections. After physeal growth modulation was performed, lesion regrowth was suppressed during more than 8 years of follow-up (18).

From a clinical management perspective, therefore, the assessment of recurrence risk depends not only on whether surgery is performed but also on thorough preoperative evaluation of lesion extent, the feasibility of complete resection, and standardized long-term postoperative imaging follow-up. In pediatric patients—particularly those with open physes and lesions located near the articular surface—special attention should be paid to the possibility of residual lesions and subsequent regrowth. Based on the present case and previous literature, we propose the following follow-up strategy: imaging examinations (such as MRI, radiography, or CT) at 1, 3, 6, and 12 months postoperatively. As this disease most commonly involves the epiphysis, the 1-month follow-up should primarily focus on recovery of postoperative joint function. The 3- and 6-month evaluations should emphasize the detection of early signs of recurrence, whereas the 12-month assessment may serve as the final follow-up. This proposed strategy is based on clinical experience; due to the rarity of this condition, it has not yet been validated in large clinical cohorts.

## Limitations

The primary limitation of this study is that postoperative follow-up was conducted exclusively through telephone interviews, without objective clinical examinations or imaging reassessments. Although the child’s family reported no apparent postoperative pain, this information is insufficient to accurately evaluate ankle joint functional recovery, osteoarticular development, or long-term outcomes. In addition, because the family declined further outpatient visits and imaging follow-up, continuous longitudinal data were unavailable, limiting our ability to perform an in-depth analysis of the disease course after recurrence and the effectiveness of treatment. As this is a single-case report, the findings require validation through additional cases and long-term follow-up studies.

## Conclusion

This study reports a rare case of recurrent DEH and presents a systematic review of the literature from the past decade focusing on its diagnosis, differential diagnosis, treatment, and follow-up. The findings suggest that accurate diagnosis of DEH requires comprehensive evaluation integrating clinical presentation, sequential imaging findings, and pathological examination, with particular attention to distinguishing it from conditions such as osteochondroma. In recurrent cases, assessing the feasibility of complete excision and implementing individualized follow-up management are clinically important. This study may provide a reference for the standardized identification of DEH and support clinical decision-making as well as the development of standardized follow-up strategies.

## Data Availability

The original contributions presented in the study are included in the article/Supplementary Material, further inquiries can be directed to the corresponding authors.
