# Supplementary material for: Recurrent dysplasia epiphysealis hemimelica: a case report and review of the literature
Source: Front Med (Lausanne). 2026 May 25;13:1750247. doi: 10.3389/fmed.2026.1750247 (PMC13243120; doi:10.3389/fmed.2026.1750247)
Supplement: Supplementary file 1 [file Data_Sheet_1.PDF]

CARE case report guidelines

CARE Checklist of information to include when writing a case report

(cc) BY-NC-ND

Topic	Item	Checklist item description	Reported on Line
Title	1	The diagnosis or intervention of primary focus followed by the words "case report"	Line1/Page1
Key Words	2	2 to 5 key words that identify diagnoses or interventions in this case report, including "case report"	Lines15-16/Page1
Abstract (no references)	3a	Introduction: What is unique about this case and what does it add to the scientific literature?	Lines27-34/Page1
	3b	Main symptoms and/or important clinical findings	Lines28-30/Page1
	3c	The main diagnoses, therapeutic interventions, and outcomes	Lines30-31/Page1
	3d	Conclusion—What is the main "take-away" lesson(s) from this case?	Lines31-34/Page1
Introduction	4	One or two paragraphs summarizing why this case is unique (**may include references**)	Lines36-62/Pages1-2
Patient Information	5a	De-identified patient specific information.	Lines64-70/Pages2-3
	5b	Primary concerns and symptoms of the patient.	Lines83-84/Page3
	5c	Medical, family, and psycho-social history including relevant genetic information	Lines64-70/Pages2-3
	5d	Relevant past interventions with outcomes	Lines73-87/Page3
Clinical Findings	6	Describe significant physical examination (PE) and important clinical findings.	Lines82-90/Page3
Timeline	7	Historical and current information from this episode of care organized as a timeline	Lines71-113/Page3-4
Diagnostic Assessment	8a	Diagnostic testing (such as PE, laboratory testing, imaging, surveys).	Lines82-87,109-113/Page3-4
	8b	Diagnostic challenges (such as access to testing, financial, or cultural)	Lines191-205/Pages15-16 Di
	8c	agnosis (including other diagnoses considered)	Lines87-90/Page3
	8d	Prognosis (such as staging in oncology) where applicable	Not applicable
Therapeutic Intervention	9a	Types of therapeutic intervention (such as pharmacologic, surgical, preventive, self-care)	Lines89-90/Page3
	9b	Administration of therapeutic intervention (such as dosage, strength, duration)	Lines100-108/Page4
	9c	Changes in therapeutic intervention (with rationale)	Lines71-90/Page3
Follow-up and Outcomes	10a	Clinician and patient-assessed outcomes (if available)	Lines114-127/Pages4-5
	10b	Important follow-up diagnostic and other test results	Lines125-127/Page4
	10c	Intervention adherence and tolerability (How was this assessed?)	Lines114-127/Pages4-5
	10d	Adverse and unanticipated events	Lines114-124/Page4
Discussion	11a	A scientific discussion of the strengths AND limitations associated with this case report	Lines313-332/Pages19-20
	11b	Discussion of the relevant medical literature **with references**.	Lines350-477/Page20-23
	11c	The scientific rationale for any conclusions (including assessment of possible causes)	Lines216-312/Pages16-19
	11d	The primary "take-away" lessons of this case report (without references) in a one paragraph conclusion	Lines324-332/Page20
Patient Perspective	12	The patient should share their perspective in one to two paragraphs on the treatment(s) they received	Lines114-127/Pages4-5
Informed Consent	13	Did the patient give informed consent? Please provide if requested	Yes ☑ No ☐
