# Supplementary material for: Recurrent dysplasia epiphysealis hemimelica: a case report and review of the literature
Source: Front Med (Lausanne). 2026 May 25;13:1750247. doi: 10.3389/fmed.2026.1750247 (PMC13243120; doi:10.3389/fmed.2026.1750247)
Supplement: Supplementary file 2 [file Data_Sheet_2.PDF]

1.wos:Dysplasia Epiphysealis Hemimelica (Topic) OR Trevor disease (Topic) AND csae report (Topic) and 2025 or 2024 or 2022 or 2023 or 2021 or 2020 or 2019 or 2018 or 2017 or 2016 (Publication Years) 31

2.Cochrane library:Pubmed:((Dysplasia Epiphysealis Hemimelica) OR (Trevor disease)) AND (case report) 2016-2026 38

3."dysplasia epiphysealis hemimelica" in Title Abstract Keyword OR "Trevor's disease" in Title Abstract Keyword AND "case report" in Title Abstract Keyword 0
